# Influence of maternal breast milk ingestion on acquisition of the intestinal microbiome in preterm infants

**DOI:** 10.1186/s40168-016-0214-x

**Published:** 2016-12-30

**Authors:** Katherine E. Gregory, Buck S. Samuel, Pearl Houghteling, Guru Shan, Frederick M. Ausubel, Ruslan I. Sadreyev, W. Allan Walker

**Affiliations:** 1 Department of Pediatric Newborn Medicine, Brigham and Women’s Hospital, 75 Francis Street, Boston, MA 02115 USA; 2 Alkek Center for Metagenomics and Microbiome Research, Department of Molecular Virology and Microbiology, Baylor College of Medicine, Houston, TX 77030 USA; 3 Department of Pediatrics, Yale School of Medicine, New Haven, CT USA; 4 Cooper Medical School, Camden, NJ USA; 5 Department of Molecular Biology, Massachusetts General Hospital, Department of Genetics, Harvard Medical School, Boston, MA USA; 6 Department of Molecular Biology, Massachusetts General Hospital, Department of Pathology, Harvard Medical School, Boston, MA USA; 7 Department of Pediatrics, Mucosal Immunology and Biology Research Center, Massachusetts General Hospital for Children, Harvard Medical School, Boston, MA USA

**Keywords:** Preterm infant, Intestinal colonization, Microbiome, Breast milk, Nutrition, Newborn intensive care

## Abstract

**Background:**

The initial acquisition and early development of the intestinal microbiome during infancy are important to human health across the lifespan. Mode of birth, antibiotic administration, environment of care, and nutrition have all been shown to play a role in the assembly of the intestinal microbiome during early life. For preterm infants, who are disproportionately at risk of inflammatory intestinal disease (i.e., necrotizing enterocolitis), a unique set of clinical factors influence the establishment of the microbiome. The purpose of this study was to establish the influence of nutritional exposures on the intestinal microbiome in a cohort of preterm infants early in life.

**Results:**

Principal component analysis of 199 samples from 30 preterm infants (<32 weeks) over the first 60 days following birth showed that the intestinal microbiome was influenced by postnatal time (*p* < 0.001, *R* ^2^ = 0.13), birth weight (*p* < 0.001, *R* ^2^ = 0.08), and nutrition (*p* < 0.001, *R* ^2^ = 0.21). Infants who were fed breast milk had a greater initial bacterial diversity and a more gradual acquisition of diversity compared to infants who were fed infant formula. The microbiome of infants fed breast milk were more similar regardless of birth weight (*p* = 0.049), in contrast to the microbiome of infants fed infant formula, which clustered differently based on birth weight (*p* < 0.001). By adjusting for differences in gut maturity, an ordered succession of microbial phylotypes was observed in breast milk-fed infants, which appeared to be disrupted in those fed infant formula. Supplementation with pasteurized donor human milk was partially successful in promoting a microbiome more similar to breast milk-fed infants and moderating rapid increases in bacterial diversity.

**Conclusions:**

The preterm infant intestinal microbiome is influenced by postnatal time, birth weight, gestational age, and nutrition. Feeding with breast milk appears to mask the influence of birth weight, suggesting a protective effect against gut immaturity in the preterm infant. These findings suggest not only a microbial mechanism underpinning the body of evidence showing that breast milk promotes intestinal health in the preterm infant but also a dynamic interplay of host and dietary factors that facilitate the colonization of and enrichment for specific microbes during establishment of the preterm infant microbiota.

**Electronic supplementary material:**

The online version of this article (doi:10.1186/s40168-016-0214-x) contains supplementary material, which is available to authorized users.

## Background

The initial acquisition and early development of the intestinal microbiome during infancy are important to human health across the lifespan [1–3]. Several factors influence the assembly of the intestinal microbiome during infancy. Mode of birth [4], antibiotic administration [5], environment of care [6], and nutritional exposures, and most notably breastfeeding [7] have all been shown to play an important role in acquisition of the intestinal microbiome. Exposure to breast milk during infancy appears to be particularly important in shaping the microbiome [8]. Among preterm infants, gestational age at birth and postnatal age at observation have also been shown to be relevant to the characteristics of their microbiome [9]. We know from clinical studies that exclusively breastfed full-term infants harbor specific health-promoting bacteria (“pioneer” bacteria) that are associated with improved immune status [8, 10]. We also know that the microbiota of breast- vs. formula-fed infants have a more profound effect on neonatal enterocyte genes that influence host protection and development [10]. What is not known is the impact of ingested expressed breast milk from mothers delivering prematurely on the composition of the preterm infant’s intestinal microbiome.

Children who are born preterm suffer a myriad of complications as a result of immature organs that are ill suited for the extrauterine environment at the time of their birth. Of the preterm infant patient population, those born prior to 32 weeks are especially vulnerable. These children require the majority of health care resources available because they are at the highest risk of neonatal morbidities, many of which have a lasting influence on health throughout childhood and across the lifespan [11, 12].

Preterm infants born prior to 32 weeks of gestation are disproportionately at risk for excessive intestinal inflammatory conditions, particularly necrotizing enterocolitis (NEC). NEC affects approximately 10% of preterm infants born less than 1500 g and is a major contributor to neonatal morbidity and mortality [13]. Preterm infants are also commonly at risk for intestinal dysbiosis that is associated with birth by cesarean section, maternal infection (e.g., chorioamnionitis), routine administration of antibiotics during the perinatal period, and a decreased exposure to maternal breast milk. In sum, intestinal dysbiosis in a preterm infant is problematic for both short- and long-term health and is thought to be a major risk factor for NEC [14].

Human breast milk is strongly recommended by the Committee on Nutrition of the European Society of Paediatric Gastroenterology, Hepatology and Nutrition as the best source of nutrition for newborn infants, including preterm infants [15]. Breast milk contains important developmental and immune-promoting factors (oligosaccharides, immunoglobulins, etc.) that are thought to protect the newborn both passively and actively against excessive intestinal inflammation [16]. Specifically, the ingestion of maternal breast milk (MBM) from mothers delivering prematurely, particularly when given as soon as possible after birth, is considered a preventative measure for the development of NEC [17, 18]. In the absence of expressed MBM, pasteurized donor human milk (PDHM) or a specialized preterm infant formula (IF) is used as the enteric source of nutrition in this population. However, neither of these latter nutritional approaches is as effective as MBM in establishing optimal immune health or in the prevention of NEC [19].

Recognizing that breast milk influences initial bacterial colonization and that MBM given to preterm infants can prevent NEC, we analyzed the influence of different forms of infant nutrition on the initial colonization process in 30 preterm infants. Few longitudinal studies evaluating the influence of nutrition on the preterm infant’s intestinal microbiome have been conducted, and to our knowledge, no study has evaluated the influence of PDHM on the early acquisition of the intestinal microbiome and how this differs from MBM and infant formula. Thus, the purpose of this study was to determine the composition of the intestinal microbiome of 30 preterm infants born <32 weeks of gestation for a period of approximately 6 weeks after birth who had been exposed to different nutritional regimens.

## Results

### Diet, maturity, and postnatal age are the greatest drivers of preterm infant microbiome composition

This study included three groups of 10 preterm infants born prior to 32 weeks of gestation (*n* = 30). The infants were grouped based on a predominant diet of MBM, PDHM, or IF. Stool samples were collected daily while the infant was hospitalized, and weekly samples were analyzed for microbiome compositional differences using 16S rDNA-based sequencing.

**Fig. 1:**
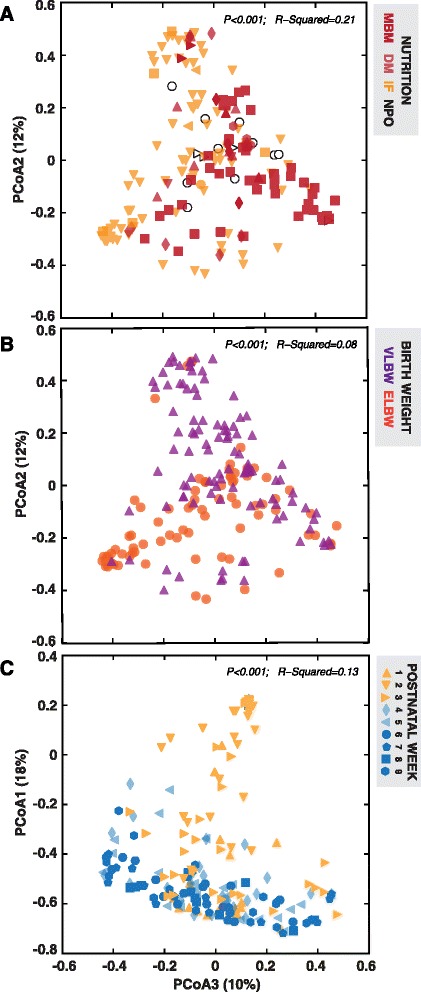
Infant microbiota composition in preterm gut driven by diet and birth weight. Principal component analysis (PCA) of 199 samples from the 30 infants over the first 60 days based on Bray-Curtis distances between samples [2000 reads/sample]. Scatterplots are colored based on the following: **a** nutritional exposures on a sample-by-sample basis [*MBM* maternal breast milk, *DM* pasteurized human donor milk, *IF* infant formula, *NPO nil per os*, nothing by mouth]; **b** birth weight class [*VLBW* very low birth weight infants 1000–1500 g, *ELBW* extremely low birth weight infants <1000 g]; and **c** week of life [*shades of yellow*—“early” samples <3 weeks; *shades of blue*—“late” samples after 3 weeks]. Early samples cluster together regardless of nutritional exposures. Closer proximity of samples (points) equals more similar microbiota composition. Separation of samples within each category is significant [FDR-adjusted PERMANOVA *p* < 0.001], and *R* ^2^ values are noted

**Fig. 2:**
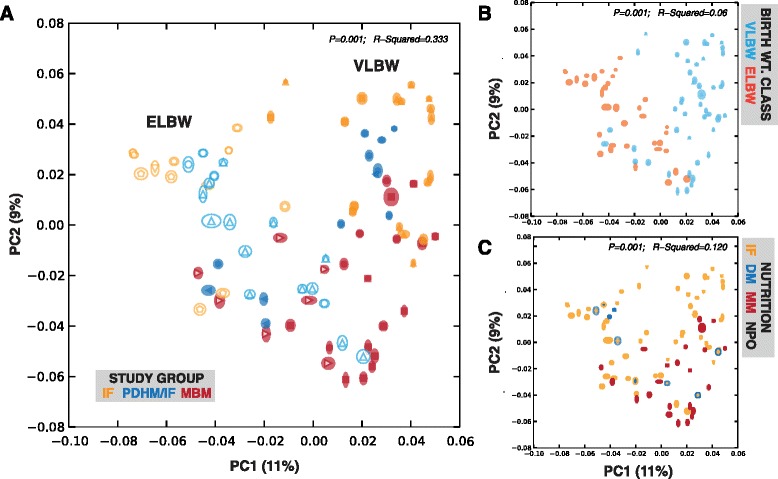
Unweighted UniFrac-based analyses of microbiomes. Clustering of samples using phylogeny-based, unweighted (“presence-absence”) UniFrac metric of microbiome composition [6965 reads/sample; ellipses for each point represent 95% confidence intervals based on jackknifed (1000×) values]. Samples are colored based on **a** study group, **b** birth weight class, and **c** nutritional exposures. Only samples with >3 weeks postnatal age are plotted [123 samples]. Separation of samples within each category is significant [FDR-adjusted PERMANOVA *p* < 0.001], and *R* ^2^ values are noted

As shown in Fig. 1, 40% of the variance in microbiome composition is explained by the first three principal components (Bray-Curtis PCA, which best explained the variance in the data among the metrics tested; see “Methods” section). In Fig. 1a, the clustering in microbiome samples based on diet are shown, where the majority of the MBM samples cluster separately from the IF samples. Figure 1b shows that the birth weight of the infant at the time of birth also contributes to the clustering of the microbiome, with infants born less than 1000 g (ELBW) grouped separately from infants born greater than 1000 g (VLBW). MBM samples cluster together regardless of birth weight, whereas IF samples formed two clusters by birth weight and gestational age. Figure 1c illustrates that postnatal week of life also appears to have a dominant role in determining microbiome composition that is largely independent of diet, as most samples after the first 3 weeks of life (“late”) clustered together along PC1 (18% of variance) separate from all of the samples within this period (“early”). This clustering is further supported based on unweighted UniFrac-based analyses of microbiome composition (Fig. 2).

### Distinct trajectories of bacterial diversity by diet

**Fig. 3:**
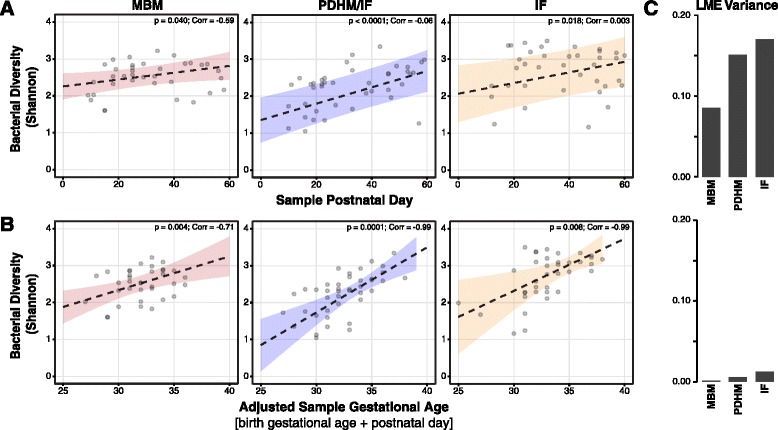
Comparison of microbial diversity trajectories by diet and maturity. Linear mixed-effects (LME) model regressions of microbial diversity (Shannon index) plotted by **a** postnatal day and **b** adjusted gestational age [gestational age + postnatal day at time of sample collection]. Adjusted gestational age reflects the overall maturity of the infant at the time of sample collection. Samples plotted included those >7 days post antibiotic exposure for both panels [118 samples]. *Lines* represent linear mixed-effects regressions (and 95% confidence intervals) of Shannon diversity over time for each infant (birth gestational age also included as a factor in panel **a**). The significance level for relating bacterial diversity over time within each diet group is noted, as are the correlations and variance (panel **c**; based on “random effects”) of the model fit

Following the initial PCA analyses that highlighted the importance of the infant’s age following birth (i.e., week of life) with regard to diet in our analyses, we sought to identify the differences in diversity (as assessed using Shannon indices; listed in Additional file 1: Table S2) over time in the microbiome between the different infant feeding groups. To account for the non-independence of the samples given that multiple samples were collected from the same infant over time, we used a linear mixed-effects modeling approach (similar to [20]) in order to control for the non-independence of the samples and account for different trajectories based on gestational age of the infants. All three nutrition groups exhibit a significant increase in bacterial diversity over postnatal time (Fig. 3a and Additional file 1: Table S3), though the groups are especially distinct with regard to the trajectories with which they increase in diversity from birth over the 60-day period. Bacterial diversity levels started more simply but rapidly increased to higher ultimate diversity levels in IF- and PDHM-fed samples compared to matched MBM-fed infant samples that exhibited a more measured initial increase in diversity. Further, variance and confidence intervals for the models of PDHM- and IF-fed infants were notably high. Adjusting for gestational age better accounted for the differences in trajectories for all nutritional groups (Fig. 3b), that is, there was a 13–579% reduction in overall variance with almost no variance (0.0001) observed in random effects models for MBM infants diversity trajectories, as well as both strong correlations to the model fit lines and much lower confidence intervals for both PDHM- and IF-fed infants (Fig. 3c). This suggests again that gestational age (or gut maturity) had as much influence on the diversity of the microbiome as any dietary regimen.

We also observed that infant formula-fed infants appear to be most susceptible to differences in gut maturity compared to MBM-fed infants. Stratification of the groups based on birth weight or birth gestational age and week of life [early <3 weeks vs. late >3 weeks] suggested that microbial diversity for both IF-fed babies and to a lesser extent PDHM-fed babies were significantly influenced by gut maturity in late samples, whereas MBM-fed babies exhibited no significant differences in bacterial diversity (Additional file 2: Figure S2).

### Succession of bacteria delayed by influence of gestational age in IF-fed infants

**Fig. 4:**
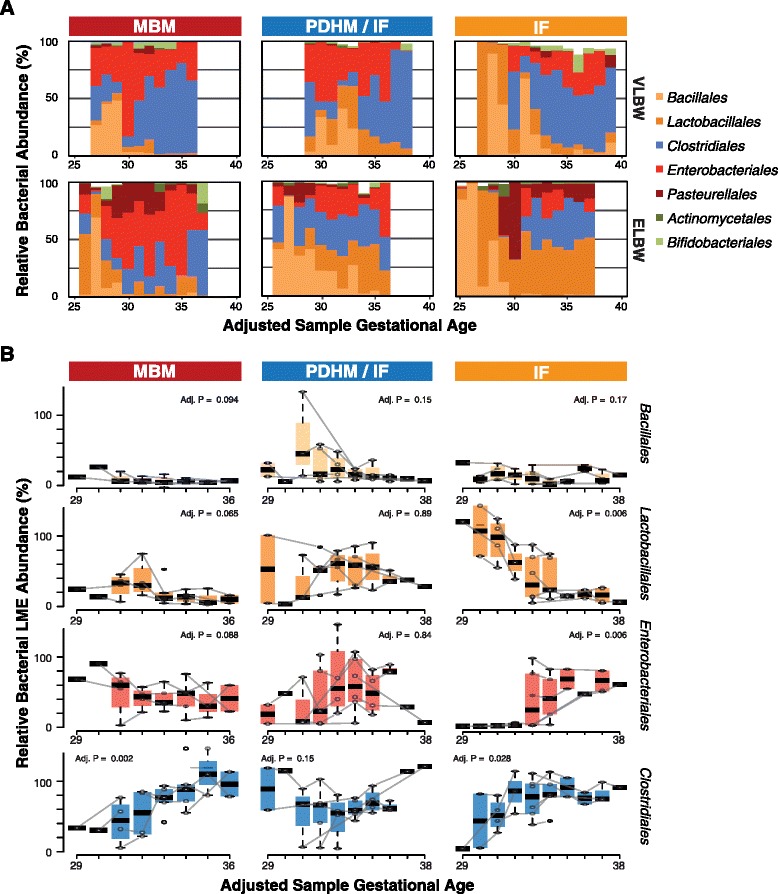
Distinct succession of bacteria as a function of diet and maturity. **a** Mean class level microbiota relative abundance in VLBW and ELBW infants by nutritional group and adjusted gestational age. **b** Linear mixed-effects (LME) modeled relative abundance and statistical analyses of the four most common types of bacteria identified: *Bacillales*, *Lactobacillales*, *Clostridiales*, and *Enterobacteriales* reported by nutritional group and adjusted gestational age [>3 weeks postnatal age, >7 days post antibiotics samples only plotted; 86 samples]. Individual infant trajectories (*lines*) for each infant are plotted along with *box* and *whiskers* for LME-modeled relative abundance values. FDR-adjusted *p* values from the LME models are noted

As seen in Fig. 4a (and Additional file 1: Table S3), the preterm infant gut is overwhelmingly comprised of species from *Bacillales* and *Lactobacillales* until approximately 28–30 weeks of adjusted gestational age, particularly in IF-fed infants, and most strikingly in IF (and to a lesser extent PDHM)-fed ELBW infants that retain high levels of *Lactobacillales* during the entire period monitored. The infants fed MBM have a greater initial diversity in their microbiome that is most strongly influenced by the presence of a variety of phylotypes that include lower levels of *Bacillales* and *Lactobacillales*, in favor of *Clostridia*, and *Enterobacteriales* as early as 26 weeks of adjusted gestational age.

We compared the overall compositional differences of the infant microbiome based on nutrition in samples obtained after 3 weeks, with no antibiotic exposure within 7 days, and fit these to a linear mixed-effects models by adjusted gestational age (see the “Methods” section; Fig. 4b). Under both MBM and PDHM conditions, we observed a relatively ordered succession in bacterial taxa with initial colonization dominated by *Bacillales/Lactobacillales* giving way to *Enterobacteriales* then *Clostridiales* and *Bifidobacteriales*. With IF feeding, a different trend emerged where higher initial and persistent levels of *Bacilliales/Lactobacillales* seemed to delay colonization by other taxa. The most striking trend was related to the *Enterobacteriales* and was not observed at appreciable levels until an adjusted gestational age of 34 weeks for IF-fed babies while MBM-fed babies had high levels of *Enterobacteriales* from the 29th week. This also in turn appeared to correlate with higher colonization levels of *Lactobacillales* and to some extent less colonization of Clostridiales. Support of the hypothesis that the delay in appearance of *Enterobacteriales* is a consequence of specific *Bacillales/Lactobacillales* inhibiting colonization of *Enterobacteriales* comes from negative correlations between particular genera within these groups (e.g., inverse Spearman correlations between *Bacillus* and *Citrobacter*; Additional file 3: Figure S3). Other differences between the groups included a more rapid increase in *Clostridiales* in the MBM and PDHM groups compared to the IF group and lower overall relative abundance of *Lactobacillales* in the MBM group compared to the IF and PDHM group. Together, these observations suggest a dynamic interplay between host and dietary selection of specific members of the microbiome that is disrupted by feeding of infant formula.

**Fig. 5:**
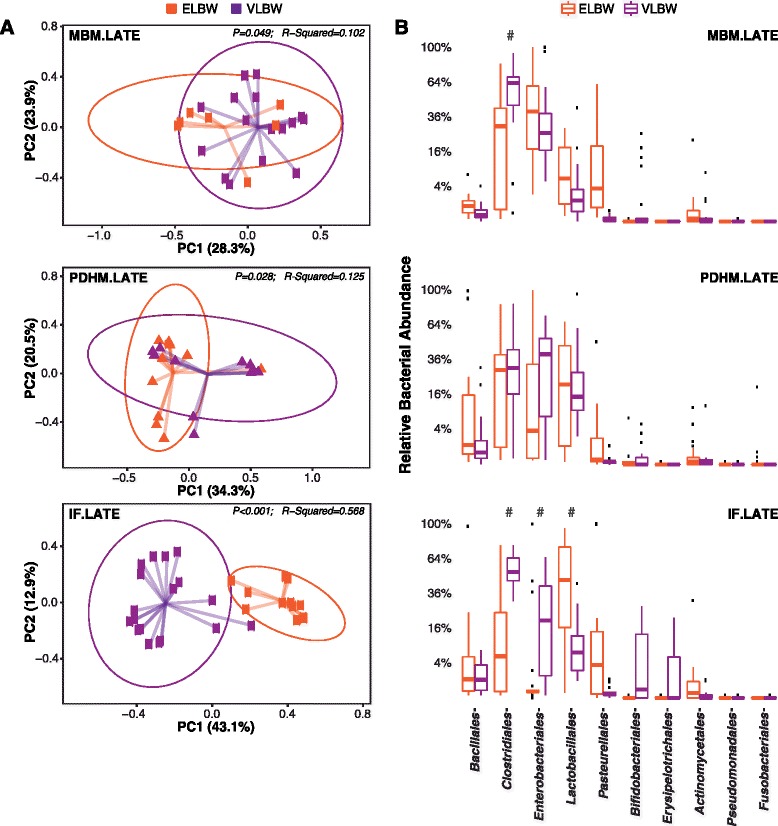
Impact of diet on microbiome composition by maturity. **a** PCA scatterplots of microbiota composition in late (>3 week) samples only by nutritional group. **b** Relative abundance of the 10 most common classes of bacteria measured. # indicate significance based on LME modeling as a function of adjusted gestational age (FDR-adjusted *p* > 0.05; Additional file 1: Table S4)

We next sought to investigate the influence, if any, of nutrition on establishment of microbiome composition by maturity of the infant at birth, as measured by gestational age at birth (Fig. 5a; samples > 3 weeks post birth). Again, the infants who were fed MBM cluster together regardless of birth weight (*p* = 0.049, *R* ^2^ = 0.102) whereas the infants fed infant formula cluster distinctly from one another based on birth weight (*p* < 0.001, *R* ^2^ = 0.568). This finding may indicate that breast milk protects against bowel immaturity associated with low birth weight, whereas in contrast, the community composition of infants fed IF can still be distinguished based on birth weight. In Fig. 5b (and Additional file 1: Table S4), we show the relative abundance of the 10 most common classes of bacteria measured in our samples. Statistically significant differences (based on the LME tests above) in the MBM group were identified *Clostridiales* (*p* = 0.002). In the IF group, statistically significant differences were identified in *Clostridiales* (*p* = 0.028), *Enterobacteriales*, and *Lactobacillales* (*p* < 0.01). In the IF-fed group, the most significant differences were identified in the *Clostridiales*, with VLBW infants having nearly ten times the amount as ELBW infants.

**Fig. 6:**
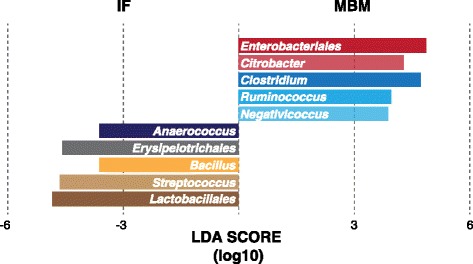
Microbes that are discriminative of infant diet. Histogram of linear discriminant analyses (LDA) effect size (LEfSe) method identified taxa that are most associated with microbiomes of MBM- and IF-fed infants. No characteristic taxa were observed within PDHM-fed infants, suggesting a more heterogeneous response that largely overlaps with either IF or MBM groups

Furthermore, we employed linear effect size feature selection (LEfSe; [21]) to identify specific taxa that were significantly associated with either MBM or IF diets (Fig. 6). *Enterobacteriales* (specifically the *Citrobacter*) and select *Clostridiales* (specifically *Clostridium*, *Ruminococcus*, and *Negativicoccus*) were identified as the best discriminators of MBM-fed infants. For IF-fed infants, *Lactobacillales* (specifically *Streptococcus*), *Bacillus*, and a distinct *Clostridiales* (*Anaerococcus*) were associated with this group. The negative correlations in taxa relative abundance observed above (Additional file 3: Figure S3) between *Citrobacter* and *Bacillus* likely reflect diet-driven differences in these infant microbiomes. Together, these analyses indicate that there are at least a handful of signature taxa that characterize each nutritional regimen once a threshold of gut maturity is achieved.

## Discussion

We know that initial colonization of the newborn gut influences the development of intestinal host defense [22, 23] and appropriate development may have a profound effect on immune health during infancy and throughout life [24]. This is in part because intestinal immune health is immature at birth and develops in conjunction with the initial colonization process. A disruption in colonization and development of intestinal host defense may account for the increase in allergy and immune-mediated morbidity (i.e., autoimmune disease) in developed countries over the last half century [25].

The host defense competency of the premature intestine is particularly immature and does not respond to initial colonization in a manner similar to full term, vaginally born, and breastfed infants [26]. For example, we have shown that premature enterocytes respond to an inflammatory stimulus with excessive inflammation and can react to commensal bacteria with higher levels of inflammation compared to mature human enterocytes [27, 28]. This suggests that dysbiosis during the newborn period of preterm infants may contribute to excessive intestinal inflammation leading to NEC.

We also know that breast milk as an exclusive source of nutrition in full-term infants stimulates health-promoting bacteria, known as “pioneer” bacteria that directly influence the development of host defense [29]. We also know that expressed breast milk from mothers delivering prematurely protects the infant from NEC [17, 18]. Although breast milk contains many passive protective factors (IgA, oligosaccharides, lactoferrin, etc.) that may contribute to the protection against inflammation and NEC, it may also provide an active role in the stimulation of health-promoting bacteria that provide protection against NEC. Several studies have suggested that probiotics given to preterm infants may also protect against NEC [30], and a recent report suggests that a combination of probiotics and breast milk may be most protective [31]. Since no studies have been conducted to determine the influence of breast milk on intestinal colonization in preterm infants, we studied the composition of intestinal microbiota in preterm infants using three standard feeding regimes: (1) exclusive ingestion of expressed MBM; (2) ingestion of PDHM followed by IF; and (3) exclusive ingestion of preterm IF. We hypothesized that ingestion of expressed MBM by preterm infants may also promote a healthy gut by the stimulation of a unique, protective intestinal microbiome and that this microbiome may provide protection against the excessive intestinal inflammation of prematurity by stimulating “pioneer” bacteria.

We found that the intestinal microbiome composition of preterm infants differed over time depending on feeding with MBM, PDHM, or IF and that these differences are also influenced by gestational age at birth and postnatal age at the time of observation. The microbiomes of infants who were fed MBM clustered separately from the infants who were fed IF after the first 3 weeks following birth. The intestinal microbiome of infants fed IF was most influenced by differences in gestational age, while the intestinal microbiome of infants fed MBM was more resilient to the influence of gestational age, following a similar trajectory regardless of the gestational age at birth or postnatal age at the time of measurement. Feeding with PDHM had a varied influence on the intestinal microbiome based on gestational age of the infant. In infants born prior to 28 weeks, PDHM did not bring the infants to an MBM-like intestinal microbiome but did influence colonization by specific taxa. Infants who were greater than 28 weeks and fed PDHM did develop a microbiome that more closely resembled that of the MBM-fed infants. These observations differ from a previous report [9] suggesting that diet and other environmental factors (i.e., the delivery mode or perinatal antibiotics use) only delayed the final composition of intestinal microbiota rather than having a meaningful influence on the assembly of the microbiome during early infancy. In this study, by addressing the influence of diet in preterm infants, we showed profound effects on the composition and diversity of the colonizing intestinal population.

When the microbial structure was examined based on diet, postnatal age at the time of sample collection, and gestational age, 40% of the variance was explained by these three factors. This suggests that feeding with MBM may mask some of the influence of birth weight and in turn, gestational age at birth. Maturation of the gut (i.e., gestational age at birth) appears to drive the trajectory of colonization of the intestinal microbiome in a nutritionally dependent manner, but only after the early colonization period that appears to occur during the first 3 weeks of life. This may represent an (noisy) establishment period for the microbiome where bona fide commensals can get a foothold before host and dietary factors select and enrich for their continued presence. Together, MBM-fed infant samples appeared to be more similar and robust in that they were less influenced by birth weight or gestational age, a proxy for gut maturity, while the converse is true for IF-fed infants where composition is greatly influenced by birth weight and gestational age.

When comparing the difference by linear mixed-effects modeling of diversity among the three groups of preterm infants fed by different means, the findings reported here suggest that preterm infants fed IF start with a less diverse, more uniform intestinal microbiome that is overwhelmingly populated by just a few types of bacteria, while the infants fed MBM have a more diverse microbiome earlier in life and as a result, a more gradual trajectory towards greater diversity over time. The presence of more bacterial species early in life, as seen in the MBM-fed infants, and the slower trajectory towards increased acquisition of diversity within the intestinal microbiome may be preferable for the preterm infant not only because a greater diversity early in life is likely to be health promoting but also because a rapid increase in microbial load may be associated with an overwhelming inflammatory response during infancy [32].

There are several factors unique to breast milk when compared to infant formula that likely contributes to the increased diversity observed in this study of preterm infants who are routinely administered antibiotics following birth. First, oligosaccharides present in breast milk provide an energy source to the intestinal microbiome, facilitating the growth of bacterial species, many of which are characterized as beneficial (i.e., commensal bacteria) to the infant gut [33, 34]. Second, breast milk contains multiple bacterial species, now referred to as the milk microbiomes [35] that actively colonize the gut. And third, breast milk contains other bioactive factors such as secretory IgA, which has been shown to alter colonization of the infant gut, protecting it against pathogenic bacteria [36, 37]. None of these factors are present in IF, presumably contributing to the lower diversity index initially observed among IF-fed preterm infants, as well as the more rapid acquisition of microbes, the majority being acquired from the hospital environment. In sum, breast milk not only modifies the environment of the infant gut to protect against pathogenic bacteria, it also facilitates colonization with commensal bacteria that promote short- and long-term immune health of the host.

Infants who receive MBM and have a greater number and diversity of bacterial species present in their intestinal microbiome do not appear to have an immune system that is overwhelmed by the acquisition of the bacteria present in their microbiome, which are being introduced from the milk or the environment. A possible explanation is that during infancy, the immune system is weakened for the purpose of acquiring intestinal bacteria [32]. The resistance to infections that is known to be suppressed during infancy may be designed to prevent overwhelming inflammation associated with the process of bacterial colonization following birth. Exposure to innate defense proteins present in MBM is another factor that is likely to mitigate intestinal inflammation and immune system activation in the setting of a more diverse intestinal microbiome during infancy [38]. For example, human milk is known to have host defense properties as a result of proteins such as lactoferrin, lysozyme, secretory IgA, IgG, secretory component, and complement C3. While these have been shown to be present at lower concentrations in milk expressed from mothers following preterm birth, they are not significantly different from that of the milk expressed following a full-term gestation [39]. These findings help explain why MBM-fed preterm infants, who we show here to have a more diverse intestinal microbiome when compared to their IF-fed counterpart, do not have an overwhelming immune response to acquisition of the microbiome following birth.

Finally, we investigated the impact of nutrition on the microbiome composition by maturity at birth. Our data provide further evidence suggesting that feeding with MBM masks the influence of varied degrees of immaturity of the gut at birth. In contrast, feeding IF does not appear to have this protective influence on the preterm infant intestinal microbiome. This finding may further explain the role that MBM plays in protecting the premature gut from NEC.

Donated milk from milk banks, which was fed to the infants in this study, undergoes complex handling following collection and prior to delivery. This typically includes two freeze thaw cycles, as well as the Holder method of pasteurization. These processes have been shown to alter the macro- and micronutrient content, as well as the bioactivity of the milk. For example, PDHM has been shown to be lower in sIgA and lactoferrin than unpasteurized milk [40, 41]. More research on the influence of milk storage and pasteurization conditions relative to the specifics of the milk microbiome and presence or absence of bioactive factors, including HMOs and other glycans, is needed to guide the optimal clinical utility of this milk.

Greater study of PDHM is needed in light of our limited understanding of the influence of storage and pasteurization conditions on the bioactive content of this milk, and importantly, whether this influences the health of the preterm infant. Our study is one of the first to explore differences in the assembly of the preterm infant intestinal microbiome following supplementation with PDHM and provides important observations regarding the influence of diet on early microbial health. Future work exploring markers of interaction between the microbiome and the host (i.e., measures of host immune response to the microbiome and metabolism) will be based on this study. Overall, we did not find supplementation with PDHM to have a striking influence on the microbiome after the infants were transitioned onto IF (this transitioned occurred by 4–5 weeks of age). We did observe some similarities among VLBW infants fed PDHM with their MBM counterparts [e.g., slower increases in bacterial diversity], but later samples resembled more often either those of IF group or a hybrid composition intermediate between the MBM and IF groups. While the infants fed PDHM did not clearly cluster together with either the “MBM microbiome” or the “IF microbiome,” there were some interesting differences based on birth weight and specific taxa present in the microbiome. For example, when accounting for gestational age, we observed an overall similar colonization levels for two of the four most abundant bacterial orders in infants fed PDHM [i.e., better colonization of *Enterobacteriales* and *Clostridiales* like MBM-fed infants] but still had considerably higher levels of *Lactobacillales* similar to IF-fed infants. This is an important finding as it suggests that for these infants, exposure to PDHM prior to IF has an influence on some members of the microbiome that mirror that of the MBM-fed infants. More research is needed, but these findings suggest that there may be some influence on the microbiome associated with early exposure to PDHM when compared to IF, perhaps as a result of specific glycans that survive the milk storage and pasteurization processes.

In this study, we analyzed specific bacteria in each feeding group. Clearly, these bacterial taxa differed between the MBM-fed and IF-fed groups. As stated, the PDHM group had an intermediate response. We then sought to determine the persistent effect of diet on microbiome composition. Again, the MBM group clustered together regardless of birth weight while the IF group was strongly influenced by birth weight. These preliminary observations indicate that mode of feeding, particularly MBM, had a specific influence on the nature of microbiota colonization in the preterm infant. Further analysis will be necessary to identify specific species and strain differences before more studies can be done.

The studies reported here suggest that expressed MBM has a significant impact on initial colonization of the preterm infant’s intestine and differs from other forms of neonatal nutrition. Moreover, this work suggests that the association of nutrition and microbiota may be key for maintaining intestinal health and in turn the prevention of NEC in this patient population. However, to demonstrate a direct association, further analyses are needed that employ a combination of isolates of specific species and in vitro techniques that we have pioneered (e.g., a fetal human small intestinal cell line (H4 cells) [42], fetal intestinal organ cultures [43], fetal intestinal xenografts [44], and organoids [45]), which could help to determine if these species specific breast milk-stimulated bacteria inhibit the intestinal inflammatory response. These observations should provide possible new probiotic protocols in combination with expressed breast milk that can be used in clinical trials and then possibly incorporated into routine care of the preterm infant.

## Conclusions

The preterm infant intestinal microbiome is influenced by postnatal time, gestational age, birth weight, and nutritional exposures. Feeding with MBM appears to mask the influence of birth weight, suggesting a protective effect against gut immaturity of the preterm infant early in life. Further study is needed regarding the influence of feeding with PDHM or IF on acquisition of the intestinal microbiome, which based on our results appears to occur in a gestational age dependent manner. These findings not only suggest a microbial mechanism underpinning the body of evidence showing that breast milk promotes intestinal health of the preterm infant, but also the dynamic interplay of host and dietary factors that facilitate the colonization of and enrichment for specific microbes during establishment of the preterm infant intestinal microbiota.

## Methods

### Study cohort and design

The infants included in this study were an otherwise healthy population of preterm infants born prior to 32 weeks of gestation. The cohort of infants was carefully assembled such that there were three groups of 10 infants that differed only on their nutritional exposures of MBM, PDHM, and IF. Inclusion in one of the three groups required that the infant be fed 100% MBM, 100% IF, or the hospital’s PDHM protocol. Within the three groups of infants, other than nutritional history, there were no statistically significant differences in gestational age, birth weight, or mode of delivery. Neonatal morbidities such as incidence of patent ductus arteriosus (PDA), sepsis, or necrotizing enterocolitis (NEC) were also used as exclusion criteria for selection of this cohort and thus were also not significantly different between the study groups. The considerations given to study design and cohort assembly in this research resulted in our ability to evaluate the influence of diet on the preterm infant intestinal microbiome over time. These results are shown in Table 1. The study design is shown in Additional file 4: Figure S1 along with accompanying sample metadata in Additional file 1: Table S1.

**Table 1:** Descriptive characteristics of preterm infants (*n* = 30)

	MBM (*n* = 10)	PDHM (*n* = 10)	IF (*n* = 10)	*p*
	Mean (SD)			
Birth weight (g)	1044 (257.6)	1070 (421.9)	1175 (348.3)	0.660
Gestational age (weeks)	28.4 (1.5)	28.4 (2.1)	28.2 (2.5)	0.971
Days to full feeds	15.10 (4.2)	16.4 (8.1)	14.45 (6.8)	0.794
First day of enteral feeding	3.8 (1.8)	5.4 (3.8)	3.9 (2.0)	0.359
	*N* (%)			
Sepsis	0 (0)	2 (20)	2 (20)	0.332
NEC				0.338
Medical	0 (0)	1 (10)	0 (0)	
Surgical	0 (0)	0 (0)	0 (0)	
Perf	0 (0)	0 (0)	0 (0)	
PDA	1 (10)	1 (10)	3 (30)	0.778
Birth mode				
Cesarean section	9 (90)	9 (90)	9 (90)	0.809

*MBM* maternal breast milk, *PDHM* pasteurized donor human milk, *IF* infant formula

### Clinical methods

All study procedures followed a protocol that was approved by the Partner’s Human Research Committee (IRB) for Brigham and Women’s Hospital and Massachusetts General Hospital (Protocol #2012-P-002453). All infants were born at Brigham and Women’s Hospital in Boston, MA, and cared for in a single-center Newborn Intensive Care Unit (NICU). Fecal samples were collected from preterm infants born prior to 32 weeks of gestation from birth until discharge or 60 days of life, whichever came first. Briefly, diapers with fecal samples were collected daily by the bedside nurse, placed in a specimen bag, and stored at 4 °C for no more than 24 h. Samples were processed daily, which involved extraction of fecal material from infant diapers using sterile procedures, and immediately frozen at −80 °C degrees until analyzed. The nutritional intake of the infants was prospectively monitored but never influenced by this observational study. When infants who were fed predominantly 100% MBM, 100% IF, or the standard hospital’s PDHM protocol were identified, their fecal samples were selected for analysis in this study. Following the hospital protocol, infants who were fed PDHM transitioned to IF after they achieved full enteral feeding (defined as >140 cc/kg/day). In addition, infants on this protocol weighing less than 1000 g at birth were required to exceed 1000 g in weight prior to transitioning from PDHM to IF.

Adjusted gestational age is calculated by adding postnatal day to gestational age at birth. For example, an infant born at 24 weeks who is now 16 days old has an adjusted gestational age of 26 weeks and 2 days, and an infant who is born at 26 weeks who is now 2 days old has an adjusted gestational age of 26 weeks and 2 days. Adjusted gestational age, sometimes called corrected age or post conceptual age, is often used to give a more accurate assessment of the overall maturity of the preterm infant and to compare across infants regardless of gestational age at birth.

### DNA extraction and sequencing

Samples were stored at −80 °C until the time of analysis. At the time of analysis, DNA was isolated from approximately 200 mg of fecal material using a commercially available kit that included a bead-beating step (MO-Bio, Carlsbad, CA). Isolated DNA from these samples (*n* = 221) was then arrayed into 96-well plates, quality determined (agarose gel), quantity normalized (by nanodrop and/or PicoGreen), and stored at −20 °C until further processing. The V4–V5 region of the 16S rRNA gene was amplified using 515 F (5′- CCTACGGGAGGCAGCAG -3′) and 806R (5′- CCGTCAATTCMTTTRAGT -3′) with unique Golay-barcoded primers (on the reverse primer) and sequencing adapters as described in the Earth Microbiome Project (http://www.earthmicrobiome.org/; [46]). To construct sequencing libraries, amplicons were purified and pooled to equimolar concentrations (PicoGreen), then sequenced using an Illumina MiSeq sequencer (paired-end 250-bp reads with v2 500 cycles kit).

The raw fastq files from sequencing were processed with the QIIME software package (v1.7.0) [47]. Briefly, sequences were truncated at the first low-quality base and quality filtered to remove reads with an average quality score below 25, reads shorter than 225 bp, reads with more than 1 ambiguous base, primer mismatches, and erroneous barcodes that could not be corrected using the Golay barcodes. Chimeric reads were removed using ChimeraSlayer [48]. The resulting dataset of quality filtered, non-chimeric of 16S rRNA gene sequences were down-sampled randomly to a maximum of 25,000 reads per sample for ease of analysis, and de novo operational taxonomic unit (OTU) picking was performed with the uclust option in QIIME [49]. Representative OTU sequences were aligned using the PyNAST algorithm with a minimum percent identity of 80% [50]. Taxonomic assignment of representative OTUs was completed using the Ribosomal Database Project (RDP) classifier [51] (greengenes 12_10). OTUs that were not identified in at least two independent samples with at least ten total reads and samples with less than 2145 reads were filtered from the dataset. 2501 OTUs were identified in the final dataset, which included 199 samples with a median of 23,309 per sample and a total of 4.1 million total reads.

For estimates of alpha diversity (within sample) and taxa relative abundance, samples were rarefied to 2145 sequences to allow for inclusion of all samples, while beta-diversity (between sample) metrics were rarefied to 6945 sequences (191 total samples) to allow for greater discrimination of microbiome composition. Alpha diversity metrics—i.e., Shannon index (H′)—were computed within QIIME using default parameters and plotted using phyloseq [52]. Principle component analyses (PCA) were completed using Bray-Curtis dissimilarity distance matrices [53] to facilitate comparisons of presence/absence patterns between samples; Bray-Curtis matrices best explained the variance of the samples (highest % variation explained by the top three PCA coordinates), though weighted and unweighted UniFrac metrics [51, 54] (among others) were also examined.

### Statistical analyses

Infant microbiome characteristics were assessed for their significance using the R software package (version 3.2.4; http://www.r-project.org/). Normally distributed variables (i.e., Shannon index) were statistically tested by an unpaired *t* test for two independent groups or a one-way analysis of variance (ANOVA) for multiple independent groups. Changes in diversity over time were evaluated by linear mixed-effects modeling in R using the nlme::lme package (v3.1-128; [55]). The infant was included as a random effect for both the intercept and the slope of the estimated fit for analyses of postnatal day and adjusted gestational age, while birth gestational age was also included in models based on postnatal days [nlme formulas − adjusted gestational age, ~1 + AdjSampleGestAge | InfantID; postnatal day, ~1 + SamplePNDay | InfantID + 1 | BirthGestAge]. Non-normally distributed variables (e.g., UniFrac, Bray-Curtis PCA) were statistically tested using PERMANOVA test followed by a Mann-Whitney *U* test for two independent groups or a Kruskal-Wallis test for multiple independent groups. Similar to diversity measures, significance of taxa relative abundance was determined using linear mixed-effects modeling as a function of adjusted gestational age using Calypso (http://bioinfo.qimr.edu.au/calypso/) [LME formula − relative taxa abundance = InfantID + AdjSampleGestAge + StudyGroup]. We sought to minimize the prevalence of zeros by summarizing taxa differences at the class level (<10% of values) and minimize the influence of the remaining zeros by transforming the datasets using arcsin square root methods (similar to [9, 56]) prior to building the LME models of relative taxa abundance. There are recent methods that explicitly address zero-inflated datasets (i.e., ZIBR [57]); however, the requirements for samples at the same timepoints in every condition were incompatible with our cohort even after imputation of missing samples [<30% of samples able to be used over a narrow range in adjusted gestational age (32–34 weeks)]. Perhaps, with a larger cohort of infants and relaxed tolerance of such datasets in future versions of the software, these limitations can be overcome.

For all analyses, the threshold significance level was set at 0.05. Corrections based on multiple testing were performed by the Benjamini-Hochberg false discovery rate procedure [58]. Linear effect size (LEfSe) analysis was performed using the default parameters to identify features that discriminated our dietary groups of interest [21].

Data including 16S rRNA gene data and metadata has been made available via the SRA database. The SRA accession number for publications referencing this dataset is SRP079978.

## Additional files

Additional file 1: Table S1. Per sample key metadata parameters. **Table S2.** Per sample Shannon diversity indices. **Table S3.** Mean taxa relative abundance values by postnatal time and birth weight class. **Table S4.** Mean taxa relative abundance values by birth weight class alone. (XLS 106 kb)
Additional file 2: Figure S2. Bacterial diversity within nutrition groups is selectively sensitive to gestational age and birth weight. Box and whisker plots of Shannon diversity indices for each of the study groups by postnatal age (<3 weeks (early) vs. >3 weeks (late)) summarized by (A) birth gestational groups [<26 weeks, 27–29 weeks and 30–32 weeks] and (B) birth weight [ELBW and VLBW]. Samples plotted included those >7 days post antibiotic exposure for both panels [118 samples]. LME models of significance as a function of diet and gestational age (*p* < 0.001) or postnatal day (*p* < 0.05) are noted in Fig. 3. (PDF 2200 kb)
Additional file 3: Figure S3. Genera co-occurrence analyses. Spearman rank co-efficient analyses of relative abundance of a given genera in all of the 199 samples as a group. Strong negative correlations between select *Enterobacteriales* [*Enterobacter*, *Trabusiella* and *Citrobacter*] and *Bacillales* [*Staphylococcus* and *Bacillus*] are shown. (PDF 2540 kb)
Additional file 4: Figure S1. Study design for longitudinal examination of nutritional exposures on preterm infant microbiome. Infants grouped by one of three feeding groups: mothers breast milk (MBM), pasteurized human donor milk (PDMH), and infant formula (IF). Samples analyzed on days indicated with an “X”. Feeding code for each day as follows: gray = NPO, red = MBM, blue = PDHM, yellow = IF. Days when a combination of feeding types were fed indicated in purple (MBM/PDHM), orange (MBM/IF), green (PDHM/IF), or brown (MBM, PDHM, IF). Birth delivery mode indicated by cesarean section (CS) or vaginal (V). Maternal antibiotics during labor and birth indicated by yes (Y). (PDF 2210 kb)
