# Supplementary figures and images for: Influence of maternal breast milk ingestion on acquisition of the intestinal microbiome in preterm infants
Source: Microbiome. 2016 Dec 30;4:68. doi: 10.1186/s40168-016-0214-x (PMC5200970; doi:10.1186/s40168-016-0214-x)

Figure S2

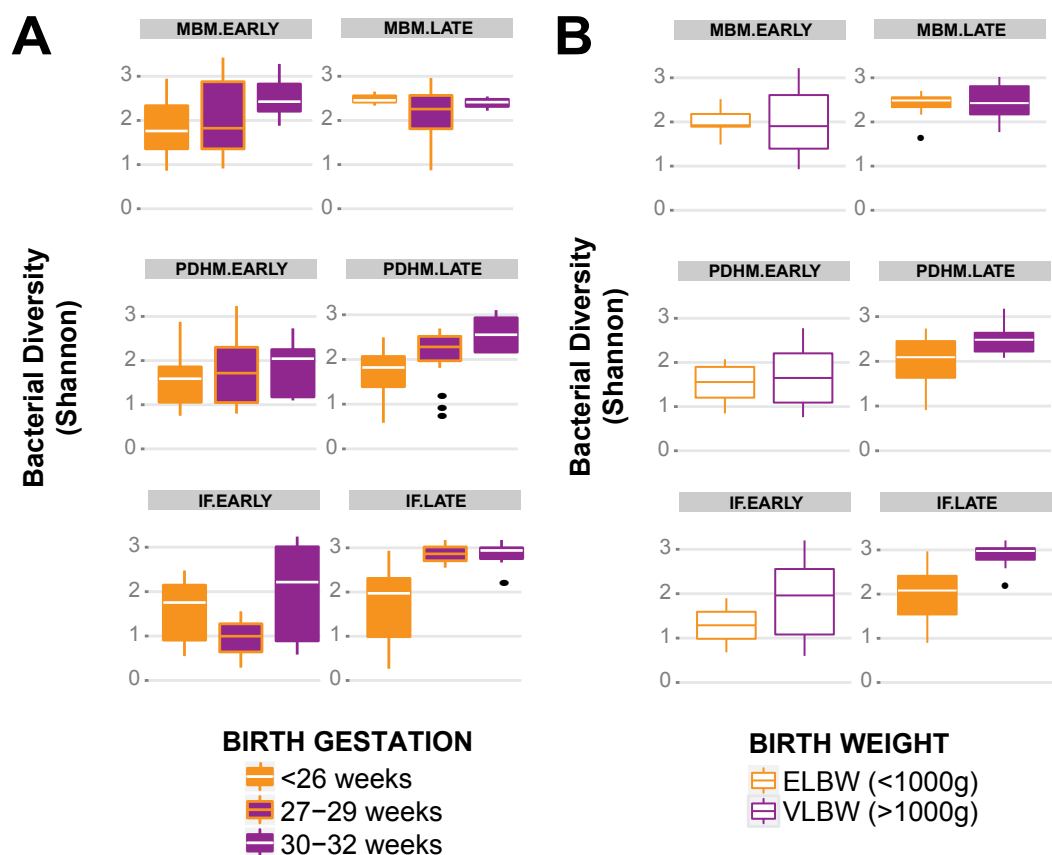

Supplement: Additional file 2: Figure S2. — Bacterial diversity within nutrition groups is selectively sensitive to gestational age and birth weight. Box and whisker plots of Shannon diversity indices for each of the study groups by postnatal age (<3 weeks (early) vs. >3 weeks (late)) summarized by (A) birth gestational groups [<26 weeks, 27–29 weeks and 30–32 weeks] and (B) birth weight [ELBW and VLBW]. Samples plotted included those >7 days post antibiotic exposure for both panels [118 samples]. LME models of significance as a function of diet and gestational age (p < 0.001) or postnatal day (p < 0.05) are noted in Fig. 3. (PDF 2200 kb) [file 40168_2016_214_MOESM2_ESM.pdf]

Figure S3

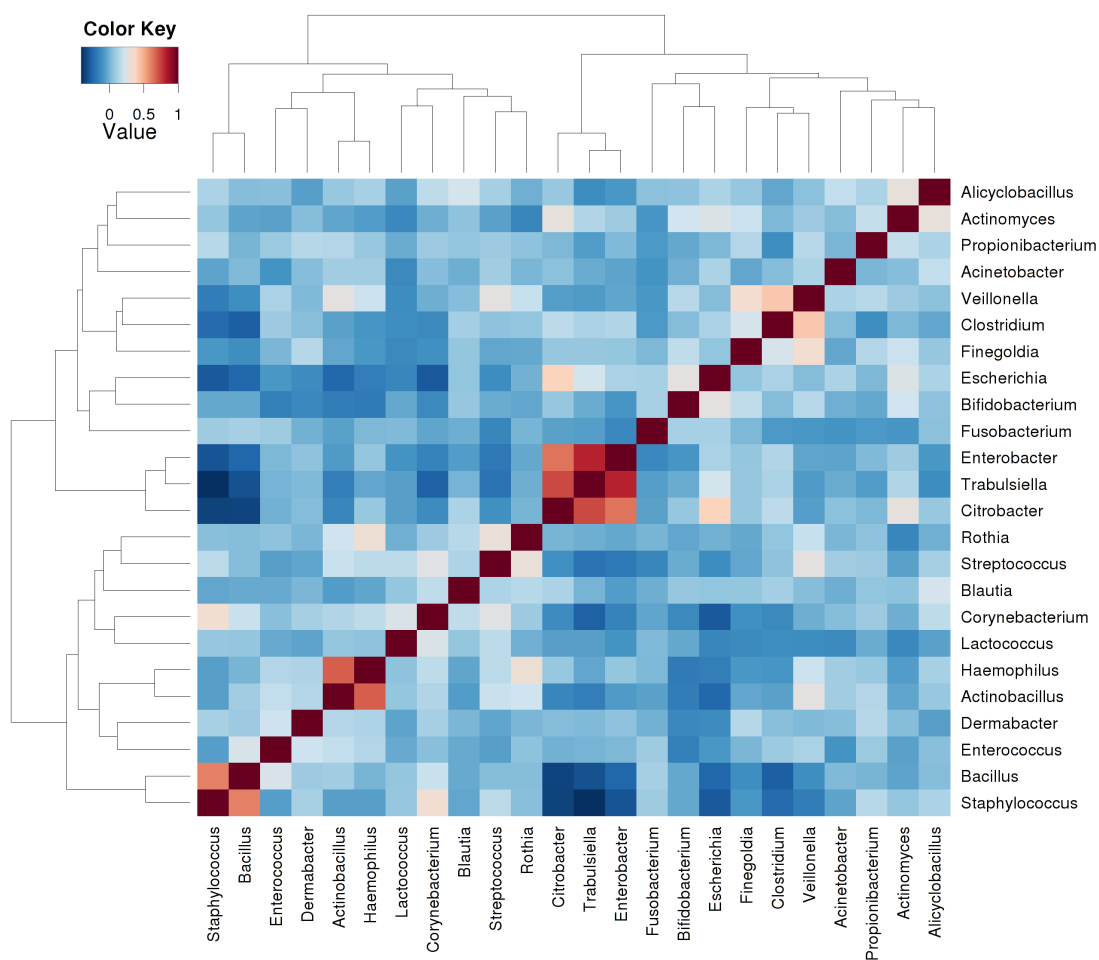

Supplement: Additional file 3: Figure S3. — Genera co-occurrence analyses. Spearman rank co-efficient analyses of relative abundance of a given genera in all of the 199 samples as a group. Strong negative correlations between select Enterobacteriales [Enterobacter, Trabusiella and Citrobacter] and Bacillales [Staphylococcus and Bacillus] are shown. (PDF 2540 kb) [file 40168_2016_214_MOESM3_ESM.pdf]

## Figure S1

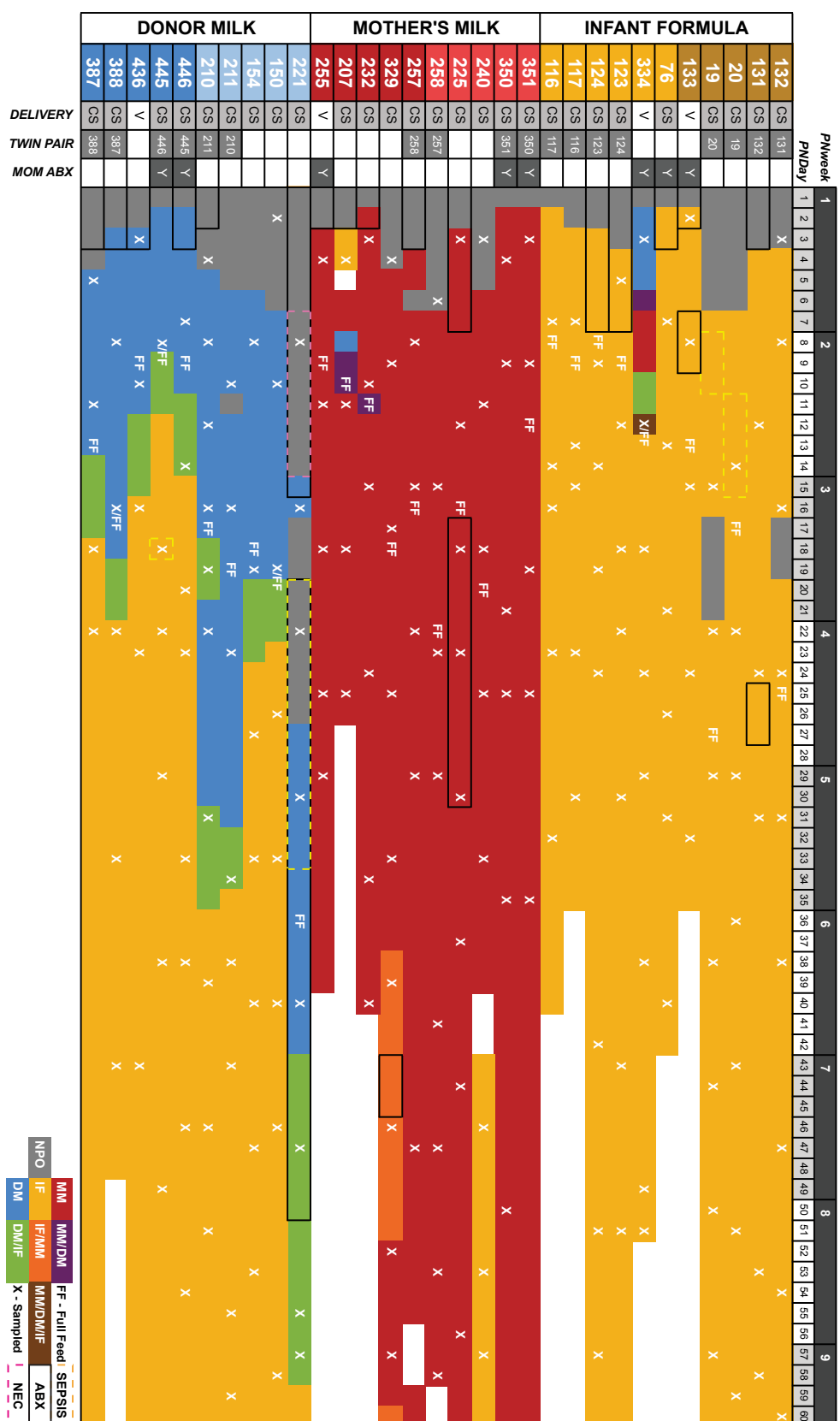

Supplement: Additional file 4: Figure S1. — Study design for longitudinal examination of nutritional exposures on preterm infant microbiome. Infants grouped by one of three feeding groups: mothers breast milk (MBM), pasteurized human donor milk (PDMH), and infant formula (IF). Samples analyzed on days indicated with an “X”. Feeding code for each day as follows: gray = NPO, red = MBM, blue = PDHM, yellow = IF. Days when a combination of feeding types were fed indicated in purple (MBM/PDHM), orange (MBM/IF), green (PDHM/IF), or brown (MBM, PDHM, IF). Birth delivery mode indicated by cesarean section (CS) or vaginal (V). Maternal antibiotics during labor and birth indicated by yes (Y). (PDF 2210 kb) [file 40168_2016_214_MOESM4_ESM.pdf]
